# Looking at the label and beyond: the effects of calorie labels, health consciousness, and demographics on caloric intake in restaurants

**DOI:** 10.1186/1479-5868-10-21

**Published:** 2013-02-08

**Authors:** Brenna Ellison, Jayson L Lusk, David Davis

**Affiliations:** 1 University of Illinois at Urbana-Champaign, 321 Mumford Hall, 1301 W. Gregory Dr., 61801, Urbana, IL, USA; 2 Oklahoma State University, 411 Ag Hall, 74078, Stillwater, OK, USA; 3 Oklahoma State University, 210 Human Sciences West, OK, 74078, Stillwater, USA

**Keywords:** Numeric vs. symbolic calorie labeling, Health consciousness, Full service restaurant

## Abstract

**Background:**

Recent legislation has required calorie labels on restaurant menus as a means of improving Americans’ health. Despite the growing research in this area, no consensus has been reached on the effectiveness of menu labels. This suggests the possibility of heterogeneity in responses to caloric labels across people with different attitudes and demographics. The purpose of this study was to explore the potential relationships between caloric intake and diners’ socio-economic characteristics and attitudes in a restaurant field experiment that systematically varied the caloric information printed on the menus.

**Methods:**

We conducted a field experiment in a full service restaurant where patrons were randomly assigned to one of three menu treatments which varied the amount of caloric information printed on the menus (none, numeric, or symbolic calorie label). At the conclusion of their meals, diners were asked to complete a brief survey regarding their socio-economic characteristics, attitudes, and meal selections. Using regression analysis, we estimated the number of entrée and extra calories ordered by diners as a function of demographic and attitudinal variables. Additionally, irrespective of the menu treatment to which a subject was assigned, our study identified which types of people are likely to be low-, medium-, and high-calorie diners.

**Results:**

Results showed that calorie labels have the greatest impact on those who are least health conscious. Additionally, using a symbolic calorie label can further reduce the caloric intake of even the most health conscious patrons. Finally, calorie labels were more likely to influence the selection of the main entrée as opposed to supplemental items such as drinks and desserts.

**Conclusions:**

If numeric calorie labels are implemented (as currently proposed), they are most likely to influence consumers who are less health conscious – probably one of the key targets of this legislation. Unfortunately, numeric labels did little for those consumers who were already more knowledgeable about health and nutrition. To reach a broader group of diners, a symbolic calorie label may be preferred as it reduced caloric intake across all levels of health consciousness.

## Background

In 1980, about 32% of food expenditures occurred outside the home. By 2010, the figure had increased to nearly 44% [1]. This increase has incited policymakers at the local, state, and national levels to push for legislation to encourage more healthful food choices away from home, with the most prominent piece being housed in the 2010 healthcare bill. This legislation mandates chain restaurants to provide calorie information on all menu forms [2]. While the intent of this type of labeling policy is quite clear, its effects are not. In a growing body of literature, a consensus on labels’ (in)effectiveness has yet to be reached – some studies found calorie labeling influenced food choice while others said it had no significant effect (see Harnack and French [3] and Swartz, Braxton, and Viera [4] for a comprehensive review).

The lack of consensus on the impacts of menu labeling suggests there may be more to the story. That previous studies have employed similar experimental designs yet reached different conclusions suggests the discrepancy may relate to differences in the types of people involved in the studies. People self-select into different types of restaurants, and it is possible menu labels are more influential for some groups of people than others. Consider health consciousness, for example. Highly health conscious individuals likely possess a large amount of health/nutrition awareness and knowledge; thus, the label will probably have minimal influence on their food choices because such individuals already know which foods are lower calorie. Low health conscious people, on the other hand, may find the label provides novel information which can be used to select a lower-calorie menu item. However, individuals (even health conscious dietitians) often struggle to estimate (typically underestimate) the number of calories in restaurant meals [5-7]. Thus, when diners are confronted with accurate calorie information, their attitudes toward specific menu items may change, especially for items not closely aligned with expectations. Burton et al. [7] argue “surprising” menu items (i.e., a high-calorie salad) will experience the most dramatic shifts in attitudes and purchase intentions. Differences in conclusions across studies might partially be explained by the fact that “surprises” may differ across people and restaurants

The impact of menu labels may also vary with demographic factors, such as gender, income, age, and education. Glanz et al. [8] found that nutrition is more important to women and older individuals; thus, these groups may be more responsive to menu labels as opposed to young males. Surprisingly, the menu labeling literature has largely neglected the impacts of demographics and attitudinal characteristics. There have been several studies on the types of people who eat at fast food restaurants (see Rydell et al. [9] for a review), but little work has examined what people eat once inside the restaurant, a gap the present study aims to fill.

In this paper, we also investigate the effect of the format in which calories are displayed on menu labels. The vast majority of labeling studies have provided the number of calories for each menu item. From the literature, it is clear this type of label has limited effectiveness, which leads us to ask: is there a better way to convey caloric information? Thorndike et al. [10] found using a traffic light symbol adjusted purchasing behavior among hospital cafeteria patrons; however, there was no comparison with other labeling formats. Alternatively, Ellison, Lusk, and Davis [11] compared the effectiveness of symbolic (also in the form of a traffic light) versus numeric menu labeling and found that symbolic labeling led to lower caloric intake, on average, than numeric labeling. An open question this study aims to answer is whether symbolic information might be more influential on consumers with limited nutrition knowledge.

The overall purpose of this study is to gain a better understanding of restaurant patrons’ choices in the face of differing nutrition labels. More specifically, we will determine which types of people are most responsive to nutrition labeling on restaurant menus by examining the relationship between caloric intake and (1) menu labeling format, (2) health consciousness, and (3) demographic factors.

## Methods

### Data and experimental design

Survey data were collected for two weeks during the 2010 Fall semester at a restaurant on the Oklahoma State University campus. ^1^ The restaurant was split into three sections, with each assigned to a unique menu treatment. Upon arrival, diners were randomly assigned to a table in one of the three sections. All treatments listed the name, description, and price for each menu item, but the caloric information differed across treatments. Diners in the control menu treatment received no nutritional information, patrons in the calorie-only menu treatment were provided the number of calories in parentheses before each item’s price, and individuals in the calorie+traffic light menu treatment were presented with a green, yellow, or red traffic light symbol (indicating specific calorie ranges) in addition to the numeric caloric information preceding each item’s price. Green light options contained 400 calories or less, yellow light options had between 401 and 800 calories, and red light options consisted of more than 800 calories.

Diners could choose from 51 menu options. Major menu categories included soups and salads, burgers and sandwiches, pasta, vegetarian items, and prime and choice steaks. Additionally, diners had the option of a daily special, usually a ‘surf-and-turf’ combination. Upon completion of their meal, patrons were asked to complete a survey. Prior to this point, diners were unaware their dining choices had been recorded as part of the research study. Using the restaurant’s record-keeping system, we matched up diners’ actual choices with their survey responses. In total, there were 138 observations (see Table 1 for summary statistics).

**Table 1:** **Characteristics of survey respondents and definition of variables (*N*=138)**

**Variable**	**Definition**	**Mean**
Female	1 if female; 0 if male	55.8%
Student	1 if current Oklahoma State University student; 0 otherwise;	63.0%
Bachelor’s	1 if obtained bachelor’s degree; 0 otherwise	34.1%
Age1	1 if age is 18 to 34.99 years; 0 otherwise	69.6%
Age2	1 if age is 35 to 54.99 years; 0 otherwise	18.1%
Age3	1 if older than 55 years of age; 0 otherwise	12.3%
Income1	1 if annual household income is less than $25,000; 0 otherwise	44.2%
Income2	1 if annual household income is between $25,000 and $99,999; 0 otherwise	39.9%
Income3	1 if annual household income is $100,000 or greater; 0 otherwise	15.9%
Health Consciousness (HC)	Level of health consciousness (can range from 3 to 15)	10.319
Value Taste	1 if taste is most important characteristic in meal selection;0 otherwise	72.5%
Value Health	1 if healthfulness is most important characteristic in mealselection; 0 otherwise	10.1%
Party	Number of guests seated per table	2.928
Calorie+traffic light	1 if diner received calorie+traffic light menu; 0 otherwise	39.1%
Calorie-only	1 if diner received calorie-only menu; 0 otherwise	39.1%
Control	1 if diner received control menu with no nutritionalinformation; 0 otherwise	21.7%
Repeat Visitor	1 if diner is repeat visitor to the restaurant; 0 otherwise	61.6%
Lunch with Friends	1 if occasion for eating is lunch with friends; 0 otherwise	63.8%
Business Lunch	1 if occasion for eating is business or work-related;0 otherwise	18.8%
Entrée Calories	Main entrée calories ordered per diner	606.341
Extra Calories	Extra calories beyond main entrée (i.e., additional sideitems, desserts, drinks) ordered per diner	152.174
Total Calories	Total calories ordered per diner	758.515

The one-page survey contained 15 questions and asked about diners’: (1) demographic characteristics, (2) levels of health consciousness, (3) frequency of and reasons for dining at the restaurant, (4) method of item selection (i.e., was selection based on taste, price, healthfulness, etc.), and (5) menu label preference. On the back of the survey, participants were presented a menu and asked which item(s) and beverage they ordered and if they ordered dessert (see Additional file 1).

A key variable in this analysis was health consciousness. Following Kraft and Goodell [12] and Berning, Chouinard, and McCluskey [13], we measured this construct by asking participants to answer three five-point Likert scale questions regarding their daily caloric intake, fat intake, and use of nutrition labels. Summing the values across the three questions provided a person’s level of health consciousness; scores could range from three to fifteen, with fifteen representing the most health conscious consumer.

### Model and data analysis

The first part of our analysis utilized ordinary least squares (OLS) regressions to determine factors affecting diners’ caloric intake. We disaggregated total caloric intake into (1) main entrée calories consumed, and (2) extra calories derived from additional items consumed over the course of the meal (drinks, desserts, side items like soup or salad served before the main course, etc.). Some extra items (namely, daily dessert specials and drinks) were not listed on the menu, and in concordance with the new federal labeling law, were thus not required to possess a menu label. ^2^ The model for calorie intake type *m* (*m* = entrée calories, extra calories) by individual *i* is specified as follows:

(1) $$\begin{array}{l} {\text{CI}}_{i}^{m}=\beta_{0}^{m}+\beta_{1}^{m}{\text{TLS}}_{i}+\beta_{2}^{m}{\text{CAL}}_{i}+\beta_{3}^{m}{\text{HC}}_{i}\\ \;+\beta_{4}^{m}{\text{Female}}_{i}+\beta_{5}^{m}{\text{Student}}_{i}\\ \;+\beta_{6}^{m}{\text{Bachelors}}_{i}+\beta_{7}^{m}{\text{Party}}_{i}\\ \;+\gamma_{1}^{m}{\text{TLS}}_{i}{*\text{HC}}_{i}+\gamma_{2}^{m}{\text{CAL}}_{i}{*\text{HC}}_{i}+\varepsilon_{i} \end{array}$$

where *β*_0_ is the intercept, *β*_1_, …, *β*_7_ are the effects of the calorie+traffic light (*TLS*_*i*_) and calorie-only (*CAL*_*i*_) menu labeling formats, health consciousness (*HC*_*i*_), gender (*Female*_*i*_), status as a current student (*Student*_*i*_), college education (*Bachelors*_*i*_), and party size (*Party*_*i*_) on caloric intake, *γ*_1_ and *γ*_2_ are interaction effects between each menu labeling format and health consciousness on caloric intake, and *ε*_*i*_ ~ *N*(0, *σ*_*ε*_^2^) is a random error term.

Despite mixed results from previous studies, we hypothesized lower caloric intake among those individuals who received menus providing nutritional information (the calorie+traffic light and calorie-only menus) compared to those individuals who received no nutritional information (i.e., *β*_1_ < 0 and *β*_2_ < 0). Research has shown consumers tend to underestimate the caloric contents of meals [5-7,14], so the label corrects the misperception and may lead to lower-calorie choices. Additionally, we expected these negative relationships to hold more strongly in the entrée calorie specification as opposed to the extra calorie specification since some extra calorie items (drinks and desserts) were not included on the menu.

Secondly, we hypothesized a negative relationship between health consciousness and caloric intake. The more health conscious a person is (i.e., the more a person monitors his/her calorie and/or fat intake or spends time reading nutrition labels), the greater amount of nutrition knowledge/awareness the individual has, and thus, the fewer calories that individual is expected to order. However, we expected high levels of health consciousness will moderate the effect of menu labeling format such that highly health conscious individuals will derive little new information from calorie labels. Thus, we hypothesized that menu labeling format will lead to the greatest calorie reductions for individuals who were less health conscious.

In the second portion of our analysis, we focused on answering the “who orders what” question. Here, we determined which types of people (male vs. female, older vs. younger, etc.) were low-, medium-, and high-calorie diners. For this, we again considered both entrée and extra calories ordered; however, instead of examining them as continuous variables, we segregated people into low, medium, and high categories. For the entrée calories, we used the intuitive cutoff points corresponding to our traffic light specifications. Thus, low-calorie diners ordered 400 entrée calories or less, medium-calorie diners ordered between 401 and 800 entrée calories, and high-calorie diners ordered more than 800 entrée calories.

Defining the low, medium, and high levels of extra calories was more challenging. We opted to classify low-calorie diners as those people who ordered zero extra calories. These diners strictly adhered to their main entrée choice and did not supplement their meal. Medium-calorie diners were those who ordered between one and 250 extra calories (most likely diners who ordered one extra item), and high-calorie diners ordered more than 250 extra calories (most likely selected two or more extra items).

Once the low-, medium-, and high-calorie categories were established for entrée and extra calories, we calculated the mean values for a host of variables under each category, including gender, age, income, and education. The average levels of health consciousness were also compared across the categories of diners as well as the proportion of people who responded that taste or health was the most important characteristic when making a menu selection. A dummy variable for the menu labeling treatment was also included to determine whether one format led to more low (or even high) calorie diners than another. Finally, we included variables relating to whether individuals were repeat diners and their reason for visiting the restaurant. Chi-squared and ANOVA tests were used to determine whether significant differences existed between low-, medium-, and high-calorie diners.

## Results

**Figure 1:**
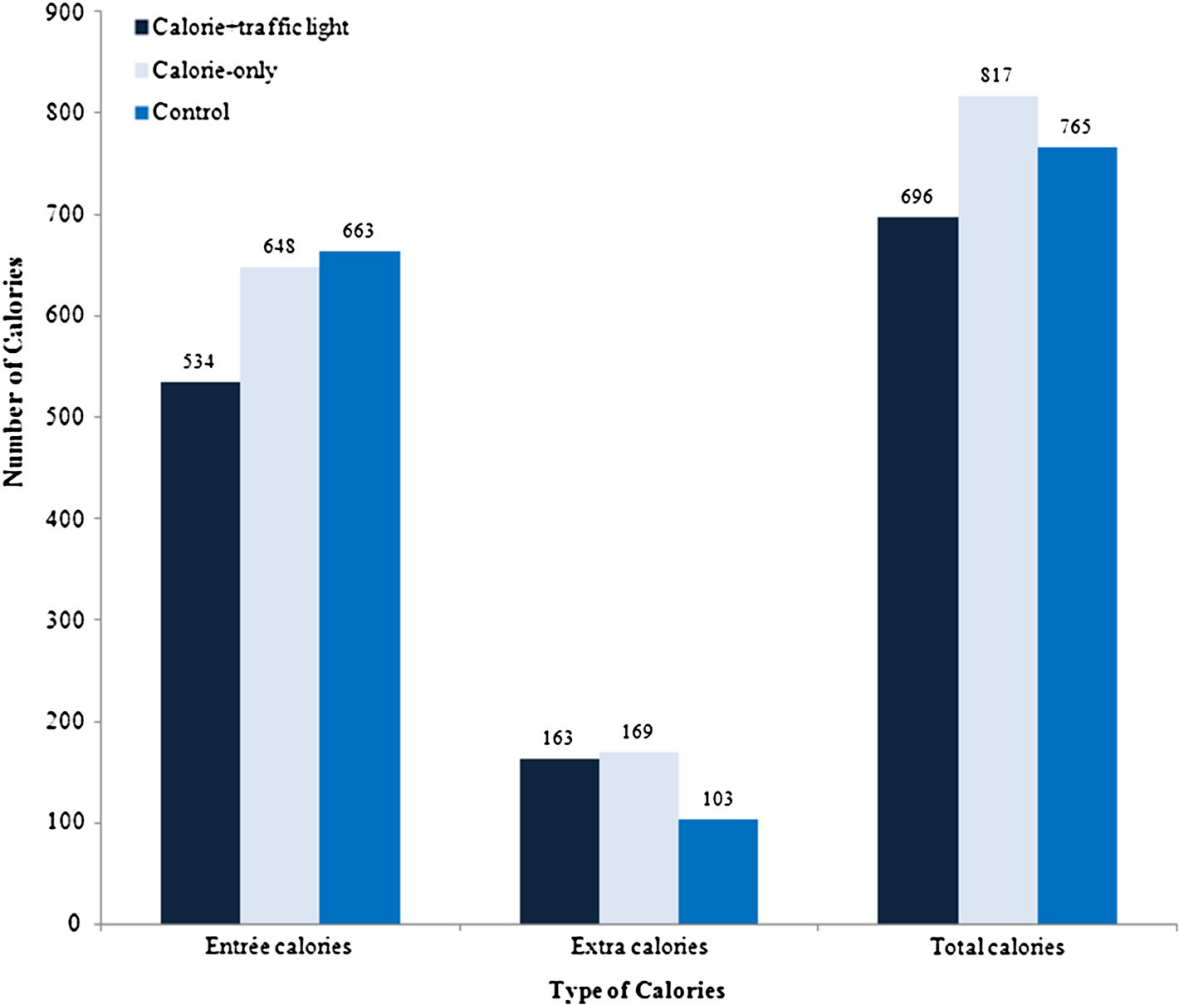
Average number of entrée, extra, and total calories across three menu treatments.

We first compared the average number of entrée, extra, and total calories ordered across the three menu formats. Figure 1 reveals that, in terms of entrée calories, the calorie-only and calorie+traffic light labeling treatments resulted in lower caloric intake relative to the control menu with no information. The calorie+traffic light menu label led to significantly fewer entrée calories ordered (p = 0.033) compared to the other two labeling formats (114 and 129 entrée calories fewer, on average, than the calorie-only and control menus, respectively). However, there were no significant differences in extra calories ordered across treatments.

Combining the entrée and extra calorie measures gave us the average total calories ordered. Ultimately, neither label significantly changed total calories ordered relative to the control menu; ^3^ however, the calorie+traffic light label outperformed the calorie-only label as these diners ordered 121 calories fewer than those receiving the calorie-only menu (p = 0.063).

### Regression analysis

**Figure 2:**
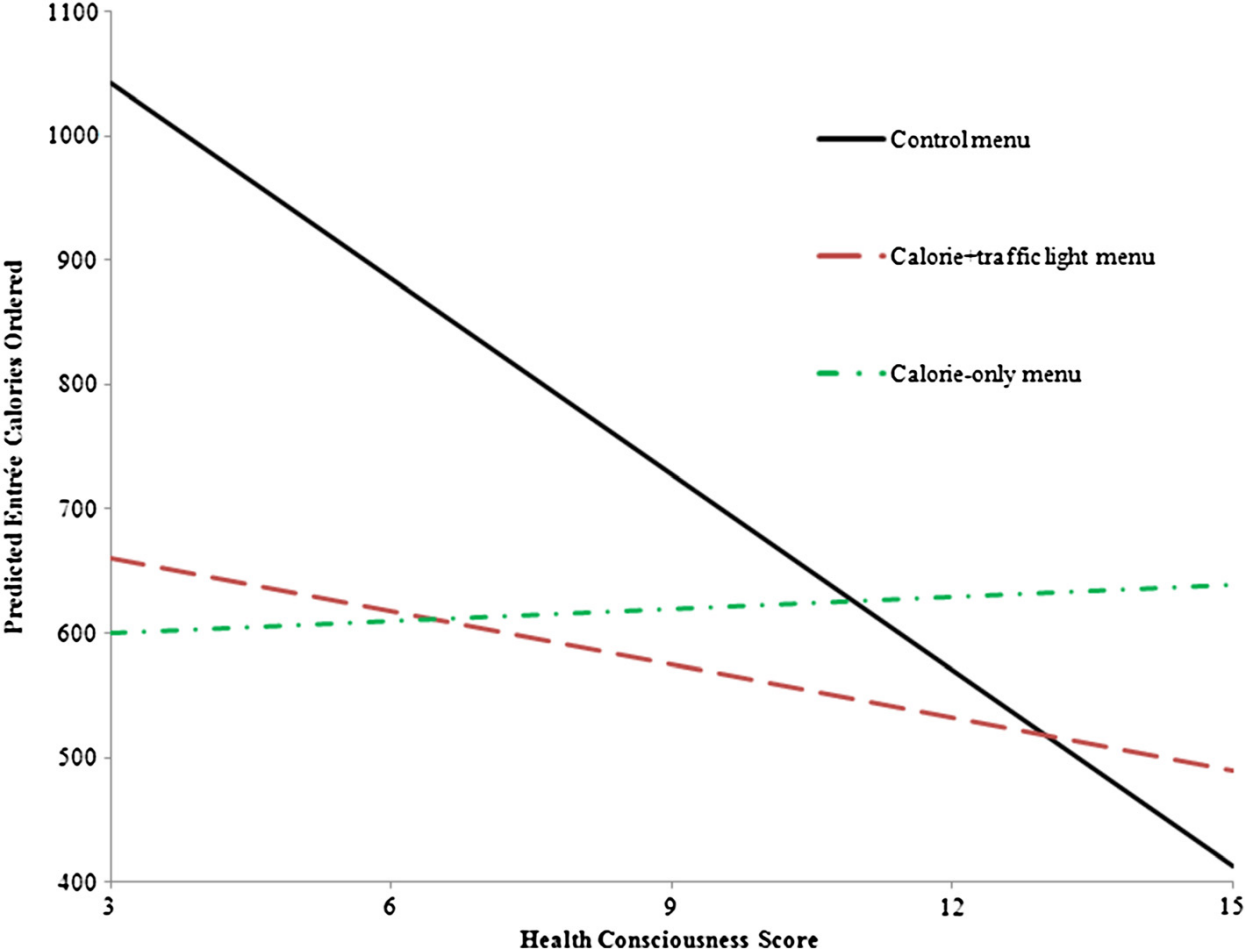
Relationship between health consciousness and entrée calories ordered in three menu treatments.

First consider the regression results for entrée calories. Table 2 shows both the calorie+traffic light and calorie-only labels significantly reduced entrée calories ordered (by 496.34 and 610.69 calories, respectively), thus *β*_1_ < 0 and *β*_2_ < 0 as hypothesized. Based on Figure 1, one might have expected the calorie+traffic light label to have the greater reduction in entrée calories; however, the interactions between each menu treatment and health consciousness must also be considered when interpreting the mean effect of a menu treatment. Table 2 reveals both interactions between menu treatment and health consciousness were significantly positive, indicating the effects of the labels were less pronounced for more health conscious individuals. Comparing the two labels, we found that at low levels of health consciousness, the calorie-only label led to larger calorie reductions; however, as health consciousness increased, the calorie+traffic light was more effective at reducing entrée calories, all else held constant. Figure 2 illustrates this effect by plotting the predicted caloric intake as a function of HC score for the three menu treatments, while holding all other variables constant at the overall means.

**Table 2:** regression estimates for entrée calories ordered and extra calories ordered

	***DV: Entrée calories***	***DV: Extra calories***
**Variable**	**Estimate**	**Estimate**
Intercept	1185.75***	456.19***
	(189.04)^a^	(144.99)
Calorie+traffic light	−496.34**	101.34
	(210.66)	(145.80)
Calorie-only	−610.69***	−77.02
	(193.01)	(140.47)
Health Consciousness (HC)	−52.48***	−15.57
	(14.93)	(9.71)
Female	−99.01**	5.12
	(40.25)	(32.23)
Student	4.82	−49.99
	(65.90)	(50.92)
Bachelor’s	−19.59	−91.91*
	(75.00)	(49.90)
Party	25.06	−39.91***
	(17.55)	(10.12)
Calorie+traffic light*HC	38.16**	−7.67
	(18.06)	(11.99)
Calorie-only*HC	55.79***	13.67
	(17.19)	(12.22)
R-Squared	0.24	0.18
Number of Observations	138	138

Note: ***, **, and * represent statistical significance at the 1%, 5%, and 10% levels, respectively.

^a^Standard errors are in parentheses (heteroskedasticity consistent standard errors).

Table 2 also reveals that entrée calories were negatively related to health consciousness (p = 0.0002). Under the control menu, every one unit increase in health consciousness resulted in a 52.48 entrée calorie decrease, on average. However, under the calorie+traffic light and calorie-only label treatments, the effects of health consciousness were less pronounced. The marginal effect of health consciousness in the calorie+traffic light treatment was −52.48 + 38.16 = −14.32, so the negative relationship continued to hold but at a lower absolute magnitude. Alternatively, in the calorie-only treatment, the marginal effect was −52.48 + 55.79 = 3.31 – effectively zero. These results suggest the calorie-only label does not really tell the most health conscious individuals any new information; therefore, entrée calories were not further reduced. Figure 2 provides further evidence of this as the calorie-only line was relatively flat across all levels of health consciousness. The calorie+traffic light label, however, appeared to provide some new information as entrée calories were further reduced in this menu condition even among more health conscious individuals.

In terms of demographics, women ordered significantly fewer (p = 0.026) entrée calories than men. This aligned with the finding by Glanz et al. [8] that nutrition was more important to women than men; thus, it is probable women will select more nutritious (lower calorie) entrées than men. A second explanation may be that women generally require fewer calories to maintain their body weight relative to men. Other demographic variables had no significant impact on entrée calories ordered.

Turning to the extra calories regression estimates, Table 2 reveals the effects of the calorie+traffic light and calorie-only labels disappeared – neither was significantly different from zero. Education, however, marginally affected (p = 0.086) extra calories ordered, as people who held a bachelor’s degree ordered 91.91 extra calories fewer, on average, than those without a degree. Additionally, party size was negatively related (p = 0.003) to extra calories ordered.

### Characteristics of low-, medium-, and high-calorie diners

Table 3 offers insight into the characteristics of low-, medium- and high-calorie diners in terms of entrée calories ordered. Table 3 shows that a significantly higher percentage (p=0.001) of females (75%) ordered low-calorie entrées compared to the percentages who ordered medium- or high-calorie entrées (56.5% and 33.3%, respectively). Additionally, current university students made up larger proportions of medium- and high-calorie diners (p = 0.100) whereas people who hold a bachelor’s degree made up a greater proportion of low-calorie diners (p = 0.099). Age also varied across categories as younger patrons (ages 18–34) were more likely to order medium- or high-calorie entrées; conversely, older patrons (ages 55 and older) were more likely to order low-calorie entrées.

**Table 3:** Demographic characteristics of low-, medium-, and high-calories diners (based on entrée calories)

**Variable**^**a**^	**Low calorie diners (≤ 400 entrée calories)**	**Medium calorie diners (401–800 entrée calories)**	**High-calorie diners (> 800 entrée calories)**
Female***	75.0%	56.5%	33.3%
Student*	50.0%	71.0%	63.9%
Bachelor’s*	47.5%	27.4%	30.6%
Age1**	52.5%	79.0%	72.2%
Age2	22.5%	16.1%	16.7%
Age3***	25.0%	4.8%	11.1%
Income1	37.5%	51.6%	38.9%
Income2	40.0%	38.7%	41.7%
Income3	22.5%	9.7%	19.4%
Calorie+traffic light	47.5%	41.9%	25.0%
Calorie-only	32.5%	38.7%	47.2%
Control	20.0%	19.4%	27.8%
Value Taste	62.5%	74.2%	80.6%
Value Health***	25.0%	4.8%	2.8%
Health Consciousness**	11.200	10.290	9.389
Repeat Visitor	70.0%	58.1%	58.3%
Lunch with Friends**	50.0%	74.2%	61.1%
Business Lunch*	30.0%	16.1%	11.1%
Number of Observations	40	62	36

Note: ***, **, and * represent statistical significance at the 1%, 5%, and 10% levels, respectively.

^a^ For variable definitions, refer to Table 1.

Individuals who considered health to be the most important characteristic when making a menu selection were more likely to be low-calorie diners (p=0.001) as opposed to medium- or high-calorie diners. Health consciousness revealed a similar result. Low-calorie diners had a mean health consciousness score of 11.2, while the mean health consciousness scores for medium- and high-calorie diners declined to 10.29 and 9.389, respectively (p = 0.046).

A final set of variables related to the reasons for eating at the restaurant. During our survey period, the top two reasons for visiting the restaurant were to have lunch with friends or some type of business/work-related meal. From the table, we see that people eating lunch with friends made up larger proportions of medium- and high-calorie diners. People visiting for business reasons were just the opposite, accounting for 30% of low-calorie diners but only 16.1% and 11.1% of medium- and high-calorie diners, respectively.

Turning to Table 4, we also categorized people as low-, medium-, or high-calorie diners based on the number of extra calories ordered. Here, the effect of gender disappeared; however, there were still differences in terms of education variables. Current university students made up greater proportions of medium- and high-calorie diners. Additionally, 47% of low-calorie diners held a bachelor’s degree compared to 13.3% and 28.6% of medium- and high-calorie diners (p = 0.004). In terms of age, 90% of medium-calorie diners were 18–34 years old (p = 0.015). Table 4 also reveals low income diners (those with < $25,000 in annual household income) made up the greatest percentages of medium- and high-calorie diners (60% and 45.2%, respectively). Alternatively, higher income patrons (those with ≥ $100,000 in annual household income) were more likely to be low-calorie diners (p = 0.024).

**Table 4:** Demographic characteristics of low-, medium-, and high-calories diners (based on extra calories)

**Variable**^**a**^	**Low calorie diners (0 extra calories)**	**Medium calorie diners (1–250 extra calories)**	**High-calorie diners (> 250 extra calories)**
Female	56.1%	56.7%	54.8%
Student*	54.5%	80.0%	64.3%
Bachelor’s***	47.0%	13.3%	28.6%
Age1**	60.6%	90.0%	69.0%
Age2*	22.7%	3.3%	21.4%
Age3	16.7%	6.7%	9.5%
Income1*	36.4%	60.0%	45.2%
Income2	39.4%	36.7%	42.9%
Income3**	24.2%	3.3%	11.9%
Calorie+traffic light	37.9%	33.3%	45.2%
Calorie-only*	34.8%	46.7%	40.5%
Control	27.3%	20.0%	14.3%
Value Taste	68.2%	76.7%	76.2%
Value Health	10.6%	3.3%	14.3%
Health Consciousness*	10.939	9.700	9.786
Repeat Visitor	63.6%	46.7%	69.0%
Lunch with Friends	56.1%	66.7%	73.8%
Business Lunch**	27.3%	6.7%	14.3%
Number of Observations	66	30	42

Note: ***, **, and * represent statistical significance at the 1%, 5%, and 10% levels, respectively.

^a^ For variable definitions, refer to Table 1.

Variables related to health had a much smaller role in classifying extra calorie diners. Health consciousness was only marginally significant (p = 0.090). Similar to the entrée calorie results, low-calorie diners had the highest health consciousness scores, on average, yet the difference in health consciousness scores across the three diner groups was much smaller.

Finally, in terms of dining purpose, we again found that patrons visiting the restaurant for business or work-related purposes were more likely to be low-calorie diners as opposed to medium- or high-calorie diners (p = 0.038).

## Discussion

The federal government passed a menu labeling law in the 2010 health care bill requiring chain restaurants to post caloric information for all menus. Increased attention to labeling laws has caused a surge in research related to the potential (and actual) effectiveness of calorie labels in restaurants. As these studies become more prevalent, one would expect the results to eventually converge on the impact of these labels; however, this has not been the case. Some studies found calorie labels significantly reduced intake while others concluded the labels had no effect. These inconclusive results led us to ask: are there factors beyond the label’s presence which influence caloric intake?

Results of this study revealed menu labels have a greater effect on entrée calories than on extra calories. Both the calorie+traffic light and calorie-only labels significantly reduced entrée calories ordered but neither significantly reduced extra calories ordered. Though not statistically significant (p = 0.294), diners who received menus with nutritional information actually ordered *more* extra calories than those who received no nutritional information. This suggests diners who received calorie information may be experiencing a licensing effect such that ordering a lower-calorie entrée gave a diner license to order an extra side item or dessert [15,16]; however, we leave this issue to future research. Another possible explanation for the label’s lack of influence on extra calories ordered could be that some of the extra items like drinks and desserts were not presented on the menu, so diners were not exposed to their caloric contents.^4^

We also found a negative relationship between health consciousness and entrée calories ordered; however, the interactions between each calorie label and health consciousness were significantly positive. This means both labels were more effective among the least health conscious – precisely the people that menu labeling laws are often trying to influence. Moreover, our results suggest the calorie+traffic light menu was more effective than the calorie-only menu at reducing entrée calories ordered as health consciousness increased.

Interestingly, despite the calorie+traffic light label’s effectiveness at reducing calories ordered, it was not the labeling format of choice. When asked which labeling format was preferred, only 27.5% of respondents wanted to see the calorie+traffic light label on their menus. Surprisingly, 42% preferred the calorie-only label which had virtually no influence on ordering behavior. These responses imply diners may want more information on their menus (the number of calories) but do not want to be told what they should or should not consume (i.e., green = good, red = bad).

A key strength of this study was the experimental design. We compared two labeling treatments to a control group with no calorie labels in a real restaurant setting. Additionally, all treatments were examined simultaneously, meaning any differences in dining habits from day to day would be picked up across all treatment groups. Secondly, this paper examined restaurant patrons more closely by administering a survey in addition to collecting purchase data.

One issue in the present study was the small sample size. While more observations are preferable, the authors have conducted a larger study comparing the same three menu labeling treatments (with purchase data only), and the effects were virtually the same [11]. In both studies, the calorie+traffic light label reduced total calories ordered by 69 calories, though the reduction was significant only with the larger sample. The calorie-only label, conversely, did not affect total calories ordered regardless of sample size.

A second limitation was that not all items (particularly drinks and desserts) were listed on the menus, so diners were not provided their caloric contents. Unfortunately, this may be a limitation consumers face even when the legislation is enacted. As currently proposed by the Food and Drug Administration, restaurants will not be required to post caloric contents for daily special items which are not regularly offered. In this study, the desserts changed daily, making them exempt from calorie labels (drinks would require labels, but restaurant management was not open to adding them to the menu in this study). Thus, while lack of calorie posting on daily special items was a limitation, our design was consistent with the proposed legislation and mirrored the reality diners are likely to encounter.

## Conclusions

Together our results suggest that calorie labels in restaurants can be effective, but only among those restaurant patrons who have lower levels of health consciousness. For highly health conscious diners, calorie labels provide little new information. However, our findings suggest the addition of a symbol (here, a traffic light symbol) to the calorie information could further reduce calories ordered, even for the most health conscious individuals.

### Endnotes

^a^All data were collected during the lunch meal (11:00 a.m. to 2:00 p.m.).

^b^Under the proposed legislation, only the daily dessert specials would be exempt from having a calorie label. Drinks would be required to be labeled; however, this restaurant did not list drinks on its menus (a feature not open to change at the time of this study), so consumers were not presented with calorie information for drink options.

^c^In the present study, we found that neither the calorie-only nor the calorie+traffic light label significantly affected total calories ordered. However, one could argue the lack of significance may be due to the small sample size (and thus, limited power) and that the reduction caused by the calorie+traffic light label (69 calorie reduction, on average) could still be significant from a public health standpoint. Fortunately, we have a larger data set (N = 946) which confirms this (see Ellison, Lusk, and Davis [11]). In the larger data set, we utilized the same three menu treatments and experimental design; however, no diner demographic and attitudinal profiles were available. Results from the larger data set showed the calorie+traffic light label leads to a nearly identical 68.7 calorie reduction (on average), a result which is statistically different than the control menu. It should be noted, though, that the calorie-only label did *not* significantly impact calories ordered in either data set.

^d^While drinks and beverages were not listed on the menu (and thus had no nutritional information present for diners), it should be pointed out that less than 25% of diners ordered either a dessert or a caloric beverage; thus, the majority of extra items ordered were listed on the menu with the corresponding nutritional information.

## Competing interests

Author disclosure: Brenna Ellison, Jayson L. Lusk, David Davis, no competing interests.

## Authors’ contributions

All of the authors were involved in designing the research. BE and JLL conducted the research and DD oversaw management of the restaurant. BE had primary responsibility for analyzing the data and writing the paper, with all of the authors contributing by reviewing and editing drafts of the manuscript. All authors read and approved the final manuscript.

## Supplementary Material

Additional file 1 Survey Instrument. Click here for file
