# Supplementary material for: Looking at the label and beyond: the effects of calorie labels, health consciousness, and demographics on caloric intake in restaurants
Source: Int J Behav Nutr Phys Act. 2013 Feb 8;10:21. doi: 10.1186/1479-5868-10-21 (PMC3598881; doi:10.1186/1479-5868-10-21)
Supplement: Additional file 1 — Survey Instrument. [file 1479-5868-10-21-S1.pdf]

QUESTIONNAIRE

1. **On average, how many times do you dine at the Rancher's Club in a month?**
   - ❑ 1-2 times/month
   - ❑ 3-4 times/month
   - ❑ 5 or more times/month
   - ❑ This is my first visit

2. **What is your reason for dining with the Rancher's Club today?**
   - ❑ Business/work-related
   - ❑ Celebratory occasion (i.e., birthday)
   - ❑ Lunch with friends
   - ❑ No specific reason

3. **What is your gender?**
   - ❑ Male
   - ❑ Female

4. **Are you currently an OSU student?**
   - ❑ Yes
   - ❑ No

5. **Do you have a Bachelor's degree from a University or College?**
   - ❑ Yes
   - ❑ No

6. **What is your age?**
   - ❑ 18-34 years old
   - ❑ 35-54 years old
   - ❑ 55 years or older

7. **What is your annual household income?**
   - ❑ Less than $25,000
   - ❑ $25,000 to $99,999
   - ❑ More than $100,000

8. **Do you agree or disagree that: I try to monitor the number of calories I consume daily?**
   - ❑ Strongly agree
   - ❑ Somewhat agree
   - ❑ Neither agree nor disagree
   - ❑ Somewhat disagree
   - ❑ Strongly disagree

9. **Do you agree or disagree that: I try to avoid high levels of fat in my diet?**
   - ❑ Strongly agree
   - ❑ Somewhat agree
   - ❑ Neither agree nor disagree
   - ❑ Somewhat disagree
   - ❑ Strongly disagree

10. **Do you agree or disagree that: I spend time looking at nutritional labels while shopping for my food?**
    - ❑ Strongly agree
    - ❑ Somewhat agree
    - ❑ Neither agree nor disagree
    - ❑ Somewhat disagree
    - ❑ Strongly disagree

11. **Do you agree or disagree that: The government should advise consumers on what to eat at restaurants?**
    - ❑ Strongly agree
    - ❑ Somewhat agree
    - ❑ Neither agree nor disagree
    - ❑ Somewhat disagree
    - ❑ Strongly disagree

12. **Do you agree or disagree that: I thought very hard about which menu item(s) I selected today?**
    - ❑ Strongly agree
    - ❑ Somewhat agree
    - ❑ Neither agree nor disagree
    - ❑ Somewhat disagree
    - ❑ Strongly disagree

13. **Do you agree or disagree that: I am good with numbers?**
    - ❑ Strongly agree
    - ❑ Somewhat agree
    - ❑ Neither agree nor disagree
    - ❑ Somewhat disagree
    - ❑ Strongly disagree

14. **Which item characteristic is *most important* when making a menu selection?**
    - ❑ Price
    - ❑ Anticipated taste
    - ❑ Healthfulness
    - ❑ Recommendations from a friend or server

15. **Next semester, the Rancher's Club will use new menus. Which menu format would you *most* like to see?**
    - ❑ Menu with no nutritional information
    - ❑ Menu with calorie contents of menu items listed
    - ❑ Menu with a symbol to represent the calorie content of menu items
